# Clinical correlates associated with the long-term response of bipolar disorder patients to lithium, valproate or lamotrigine: A retrospective study

**DOI:** 10.1371/journal.pone.0227217

**Published:** 2020-01-10

**Authors:** Young Sup Woo, Bo-Hyun Yoon, Jye-Heon Song, Jeong Seok Seo, Beomwoo Nam, Kwanghun Lee, Jonghun Lee, Young-Eun Jung, Moon-Doo Kim, Jung Goo Lee, Sheng-Min Wang, Young-Joon Kwon, Won-Myong Bahk

**Affiliations:** 1 Department of Psychiatry, College of Medicine, The Catholic University of Korea, Seoul, Republic of Korea; 2 Department of Psychiatry, Naju National Hospital, Naju, Republic of Korea; 3 Department of Psychiatry, School of Medicine, Konkuk University, Chungju, Republic of Korea; 4 Department of Psychiatry, College of Medicine, Dongguk University, Gyeongju, Republic of Korea; 5 Department of Psychiatry, School of Medicine, Catholic University of Daegu, Daegu, Republic of Korea; 6 Department of Psychiatry, School of Medicine, Jeju National University, Jeju, Republic of Korea; 7 Department of Psychiatry, Haeundae Paik Hospital, Paik Institute for Clinical Research, College of Medicine, Inje University, Busan, Republic of Korea; 8 Department of Health Science and Technology, Graduate School of Inje University, Busan, Republic of Korea; 9 Department of Psychiatry, College of Medicine, Soonchunhyang University, Cheonan, Republic of Korea; Chiba Daigaku, JAPAN

## Abstract

**Background:**

Although mood stabilizers such as lithium (LIT), valproate (VAL), and lamotrigine (LMT) appear to be efficacious treatments for bipolar disorder (BD) in research settings, the long-term response to these mood stabilizers in clinical practice is highly variable among individuals. Thus, the present study examined the characteristics associated with good or insufficient responses to long-term treatment with LIT, VAL, or LMT for BD.

**Methods:**

This study retrospectively analyzed the medical records of patients who visited an outpatient clinic with a diagnosis of BD I or II. Data from patients who were treated with one of three mood stabilizing medications (LIT, VAL, or LMT) for more than 6 months were selected, and the long-term treatment responses were evaluated using the Alda scale. For the purposes of this study, two response categories were formed: insufficient response (ISR), including non-response or poor response (Alda total score ≤ 6), and good response (GR; Alda total score ≥ 7).

**Results:**

Of the 645 patients included in the present study, 172 were prescribed LIT, 320 were prescribed VAL, and 153 were prescribed LMT for at least 6 months. A binary logistic regression analysis revealed that a diagnosis of BD II (odds ratio [OR], 8.868; 95% confidence interval [CI], 1.123–70.046; p = 0.038), comorbid alcohol/substance use disorder (OR, 4.238; 95% CI, 1.154–15.566; p = 0.030), and a history of mixed episodes (OR, 4.363; 95% CI, 1.191–15.985; p = 0.026) were significant predictors of LIT-ISR. Additionally, a depressive-predominant polarity significantly predicted LMT-GR (OR, 8.586; 95% CI, 2.767–26.644; p < 0.001).

**Conclusion:**

The present findings demonstrated that patients with a diagnosis of BD II, a comorbid alcohol/substance problem, or a history of mixed episodes were not likely to respond to LIT treatment. Additionally, LMT might be a better treatment choice for patients with a depressive-predominant polarity.

## Introduction

Bipolar disorder (BD) is a severe and common chronic illness associated with high risks of relapse and recurrence as well as increased morbidity and mortality [1, 2]. Similar to schizophrenia, BD is considered to be a condition that tends to worsen with recurrence and prolongation of the illness [3, 4]. Accordingly, BD patients with a higher number of previous episodes tend to experience higher rates of disability, greater impairments in cognitive and interpersonal functioning, and a poorer overall quality of life [5, 6]. These findings suggest that long-term maintenance treatments are of paramount importance to prevent subsequent episodes, reduce residual symptoms, and restore functioning and quality of life [7, 8].

Even though several recent BD treatment guidelines suggest first- and second-line treatments based on evidence of established long-term relapse/recurrence prevention [8, 9], clinicians often rely on a trial-and-error approach to choose from among the therapeutic alternatives. However, this approach may result in the risk of a recurrence or breakthrough [10, 11]. Thus, the prediction of long-term mood stabilization effect to specific medications could substantially reduce the risk of a breakthrough or recurrence.

The potential predictors of the long-term prophylactic response to lithium (LIT) have been widely investigated. A positive long-term prophylactic response to lithium can be predicted in patients with euphoric mania, an episodic pattern characterized by a ‘mania-depression-interval’ of the clinical course (a biphasic course in which depressive episodes occur within weeks after manias, and are then followed by illness-free intervals), an intermediate age of onset, a family history of BD, or a family history of LIT response [12, 13]. Conversely, predictors of a poor response to lithium treatment include a high number of previous hospitalizations, continuous cycling, atypical mixed or psychotic features (especially mood-incongruent psychosis), an episodic pattern of ‘depression-mania-interval’ of the clinical course, or BD II [12, 14, 15]. However, the predictors long-term prophylactic response to other agents widely used for the treatment of BD, such as valproate (VAL) or lamotrigine (LMT), remain poorly investigated.

Only a few studies have reported a relatively good response to VAL in the presence of rapid cycling course, multiple prior episodes, or comorbid substance abuse [16–20]. Likewise, few studies have observed a good response to LMT with a predominantly depressive polarity or comorbid anxiety [21, 22], or BD II [8]. However, these clinical predictors of the long-term mood stabilizing effect fail to provide conclusive evidence either in support of or in opposition to the practice [23].

Therefore, the present study used the Alda scale [13], which was designed to retrospectively assess treatment responses, to examine the demographic and clinical characteristics associated with the long-term mood stabilization effect of LIT, VAL, and LMT, three agents that are commonly recommended for the treatment of BD [24],

## Methods

### Subjects and assessments

The present study retrospectively investigated the medical records of patients who visited an outpatient clinic at one of seven investigation sites (six university hospitals and one mental hospital) from January 2017 to October 2018 with a diagnosis of BD I or II according to the DSM-IV-TR. Data were selected from patients who were age 20 or older. The data of patients who had been treated with one of the three mood stabilizing medications (LIT, VAL, or LMT) for more than 6 months were collected. Patients with insufficient data, who had a severe comorbid medical or neurological condition that could contribute to mood symptoms, who were treated with a combination of mood stabilizers and anticonvulsants (e.g., LIT, VAL, or LMT and carbamazepine, oxcarbazepine, or topiramate), and/or who had a diagnosis of a mood disorder due to a general medical condition were excluded from the analyses.

Long-term treatment responses were evaluated using the Alda scale [13], which was specifically developed to evaluate the long-term mood stabilization effect under naturalistic conditions. This scale measures the degree of improvement during the course of treatment (Criterion A), which is rated on a scale from 0–10, and weighs clinical factors that are considered to be relevant for determining whether or not the observed improvements are due to the treatment (Criteria B1-B5), which are rated as 0, 1, or 2 points. The total score on the Alda scale is obtained by subtracting the B score from the A score; any negative score (i.e., the B scale score exceeds the A scale score) is recorded as 0 [13]. Although it was originally developed to evaluate the LIT response, this scale was slightly modified for the present study by replacing the term “lithium” in the instructions and other items to the term “mood stabilizers” so that it could be administered to subjects treated with VAL or LMT as well. A psychiatrist at each patient’s initial investigation site and a psychiatrist from another investigational site independently reviewed the medical records and came to a consensus regarding the treatment response. For the purposes of this study, two response categories were formed: insufficient response (ISR), including a non-response or poor response (Alda total score ≤ 6 or less), and good response (GR; Alda total score ≥ 7; [15].

The following clinical variables that are known to affect the treatment response and the choice of treatment medication were also investigated: age; sex; marital status; age at onset; duration of illness prior to investigational medications (e.g., LIT, VAL, or LMT); family history of BD; type of BD (I or II); psychiatric and medical comorbidities, including metabolic abnormalities, predominant polarity, polarity of first episode, number of past episodes, past history of seasonal pattern, rapid cycling, psychotic symptoms, mixed episodes, and suicide attempts; and concomitant medications, including antipsychotics and antidepressants [15, 25, 26].

### Statistical analysis

The sociodemographic and clinical characteristics of the LIT, VAL, and LMT groups were compared using analysis of variance (ANOVA) and Chi-square tests. Subsequently, comparisons between subjects exhibiting a GR or an ISR were independently performed within each treatment group (LIT, VAL, or LMT). For comparisons of the demographic and clinical variables, the Chi-square test or Fisher’s exact test were used for categorical variables, and independent t-tests were used for continuous variables. Additionally, a binary logistic regression analysis with treatment response as the dependent variable and age, gender, and variables that were significant (p < 0.10) in the univariate analyses (diagnosis of BD II, comorbid alcohol/substance use disorder, recurrent episodes, and history of mixed episodes) as covariates was conducted to identify independent predictors of a GR or ISR for each treatment. All statistical analyses were performed using SPSS version 21.0, and p-values < 0.05 were considered to indicate statistical significance.

### Ethical considerations

The present study was approved by the institutional review board of Yeouido St. Mary’s Hospital in Seoul, Korea (SC16QISE0038), and was conducted according to the principles of the Declaration of Helsinki. The institutional review board also approved the exemption for informed consent because this study was a retrospective chart review.

## Results

### Sample characteristics

**Fig 1:**
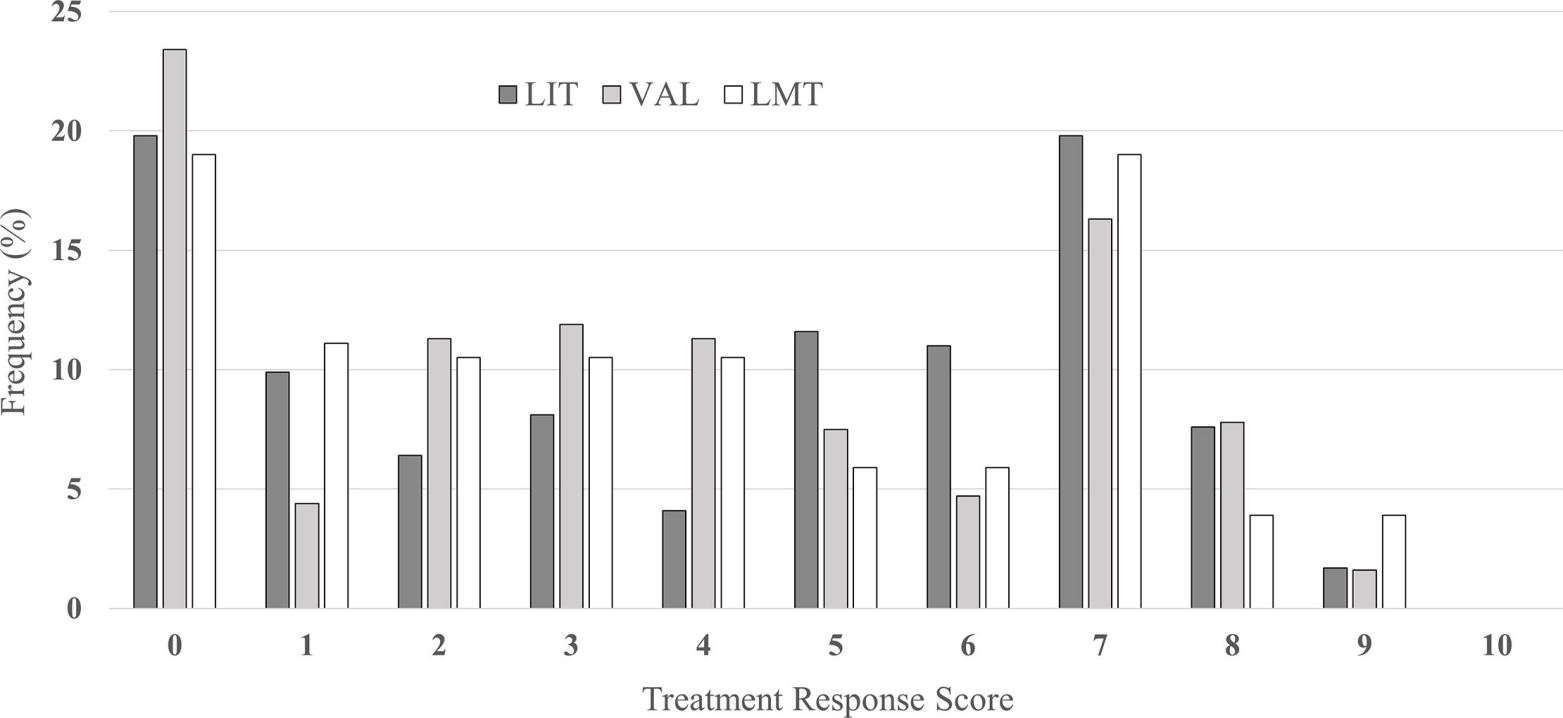
Distribution of treatment response scores for lithium, valproate and lamotrigine.

Of the 645 patients who were prescribed a mood stabilizer for at least 6 months, 172 were prescribed LIT, 320 were prescribed VAL, and 153 were prescribed LMT. Among 645 subjects, 612 (94.9%) were followed up for more than 1 year, and the median duration of follow-up was 21.6 months (range 6.0–81.6). There was no significant difference among treatment groups in duration of follow-up (p = 0.980, data not shown). The sociodemographic and clinical characteristics of the three treatment groups are summarized in Table 1. The proportions of patients with BD II, predominant manic polarity, recurrent episodes, and concomitant use of antipsychotics or antidepressants differed significantly among the groups; The order of BD II prevalence was highest in LMT group and lowest in LIT group. Patients having predominant manic polarity were highest in VAL group, followed by LIT group and LMT group. In contrast, patients having recurrent episodes or receiving concomitant antidepressants were highest for LMT group followed by LIT group and VAL group. Lastly, LIT group had the highest rate of patients receiving concomitant antipsychotics with second and third being VAL group and LMT group respectively. The distribution of treatment response scores for these three mood stabilizers is shown Fig 1 and descriptive results of treatment response scores were presented in Table 2.

**Table 1:** Comparisons of sociodemographic and clinical characteristics among treatment groups.

		Lithium group (n = 172)	Valproate group (n = 320)	Lamotrigine group (n = 153)	p-value
Age (years)		39.4±13.6	40.3±15.1	40.2±14.5	0.780
Female		85 (49.4%)	163 (50.9%)	93 (60.8%)	0.076
Elderly (≥60 years)		16 (9.3%)	36 (11.3%)	13 (8.5%)	0.600
Married		76 (44.2%)	149 (46.6%)	75 (49.0%)	0.683
Age at onset (years)		30.2±13.2	31.7±13.8	32.1±12.9	0.369
Early onset (<25 years)		69 (40.1%)	142 (44.4%)	62 (40.5%)	0.577
Family history of bipolar disorder		26 (15.1%)	50 (15.6%)	25 (16.3%)	0.955
Duration of illness until using index mood stabilizer (years)		5.1±6.1	5.1±6.3	4.8±5.6	0.849
Early life adverse event		27 (15.7%)	43 (13.4%)	21 (13.7%)	0.780
Bipolar II disorder		20 (11.6%)	55 (17.2%)	59 (38.6%)	<0.001*
Psychiatric comorbidity	Alcohol/substance use disorder	25 (14.5%)	37 (11.6%)	17 (11.1%)	0.560
	Anxiety disorder	23 (13.4%)	60 (18.8%)	23 (15.0%)	0.267
	Personality disorder	15 (8.7%)	25 (7.8%)	19 (12.4%)	0.260
Manic/hypomanic polarity at onset		106 (61.6%)	171 (53.4%)	82 (53.6%)	0.184
Predominant polarity	Manic	65 (37.8%)	125 (39.1%)	34 (22.2%)	0.001*
	Depressive	17 (9.9%)	56 (17.5%)	27 (17.6%)	0.059
Recurrent episode (≥3)		109 (63.4%)	200 (62.5%)	121 (79.1%)	0.001*
Seasonal pattern		26 (15.1%)	44 (13.8%)	23 (15.0%)	0.891
Rapid cycling		9 (5.2%)	8 (2.5%)	7 (4.6%)	0.254
Psychotic symptom		65 (37.8%)	108 (33.8%)	47 (30.7%)	0.399
Mixed episode		29 (16.9%)	53 (16.6%)	27 (17.6%)	0.957
Suicide attempt		19 (11.0%)	29 (9.1%)	24 (15.7%)	0.101
Concomitant medication	antipsychotics	144 (83.7%)	278 (86.9%)	116 (75.8%)	0.010*
	antidepressants	36 (20.9%)	62 (19.4%)	75 (49.0%)	<0.001*
Comorbid metabolic abnormalities	one or more abnormalities	47 (27.3%)	79 (24.7%)	46 (30.1%)	0.453
	diabetes mellitus	26 (15.1%)	41 (12.8%)	20 (13.1%)	0.764
	hypertension	28 (16.3%)	38 (11.9%)	25 (16.3%)	0.271
	dyslipidemia	22 (12.8%)	49 (15.3%)	25 (16.3%)	0.638

*p<0.05

**Table 2:** Total and individual criterion scores on the treatment response scale.

	Lithium group (n = 172)	Valproate group (n = 320)	Lamotrigine group (n = 153)
Total score	4.0±2.9	3.6±2.8	3.7±2.8
Criterion score			
A	6.8±2.3	6.7±2.3	7.0±1.9
B1	0.4±0.5	0.5±0.6	0.4±0.5
B2	0.4±0.6	0.5±0.6	0.4±0.5
B3	0.3±0.5	0.4±0.6	0.6±0.7
B4	0.7±0.6	0.6±0.6	0.6±0.5
B5	1.0±0.6	1.0±0.6	1.1±0.7

### Comparison of subjects with a GR or an ISR

Univariate comparisons of GR versus ISR subjects in each of the treatment groups (Table 3) revealed that the LIT-ISR subjects had significantly higher rate of BD II, an alcohol/substance use disorder, or a history of mixed episodes compared than LIT-GR subjects. Recurrent episodes were also significantly more frequent in LIT-ISR subjects than in LIT-GR subjects. Rate of BD II, an alcohol/substance use disorder, or a history of mixed episodes did not differ between the VAL-ISR and VAL-GR subjects. Although a seasonal pattern of symptoms was numerically more prevalent in VAL-GR subjects than in VAL-ISR subjects, this difference did not reach statistical significance. In the LMT group, a diagnosis of BD II, depressive predominant polarity, or recurrent episodes was more frequently observed in LMT-GR subjects than in LMT-ISR subjects. Additionally, an alcohol/substance use disorder was significantly more prevalent in LMT-ISR subjects than in LMT-GR subjects.

**Table 3:** Comparisons of sociodemographic and clinical characteristics between ISR and GR.

		Lithium group (n = 172)			Valproate group (n = 320)			Lamotrigine group (n = 153)		
		ISR (N = 122)	GR (N = 50)	significance	ISR (N = 238)	GR (N = 82)	significance	ISR (N = 112)	GR (N = 41)	significance
Age (years)		38.5±14.5	41.6±10.8	0.123	40.1±15.3	41.0±14.6	0.645	39.3±14.4	42.8±14.7	0.186
Female		64 (52.5%)	21 (42.0%)	0.213	123 (51.7%)	40 (48.8%)	0.651	66 (58.9%)	27 (65.9%)	0.437
Elderly (≥60 years)		10 (8.2%)	6 (12.0%)	0.436	27 (11.3%)	9 (11.0%)	0.927	8 (7.1%)	5 (12.2%)	0.321
Married		52 (42.6%)	24 (48.0%)	0.519	112 (47.1%)	37 (45.1%)	0.762	56 (50.0%)	19 (46.3%)	0.688
Age at onset (years)		29.5±13.6	32.0±12.3	0.258	31.9±14.0	31.4±13.3	0.790	31.6±12.9	33.6±12.8	0.384
Early onset (<25 years)		53 (43.4%)	16 (32.0%)	0.164	106 (44.5%)	36 (43.9%)	0.920	48 (42.9%)	14 (34.1%)	0.331
Family history of bipolar disorder		21 (17.2%)	5 (10.0%)	0.230	35 (14.7%)	15 (18.3%)	0.440	20 (17.9%)	5 (12.2%)	0.401
Duration of illness until using index mood stabilizer (years)		4.7±5.6	6.1±7.1	0.164	4.8±5.8	5.9±7.4	0.193	4.3±4.6	6.0±7.7	0.104
Early life adverse event		20 (16.4%)	7 (14.0%)	0.695	33 (13.9%)	10 (12.2%)	0.702	18 (16.1%)	3 (7.3%)	0.163
Bipolar II disorder		19 (15.6%)	1 (2.0%)	0.012*	42 (17.6%)	13 (15.9%)	0.710	37 (33.0%)	22 (53.7%)	0.020*
Psychiatric comorbidity	Alcohol/substance use disorder	22 (18.0%)	3 (6.0%)	0.042*	25 (10.5%)	12 (14.6%)	0.313	17 (15.2%)	0	0.008*
	Anxiety disorder	19 (15.6%)	4 (8.0%)	0.185	44 (18.5%)	16 (19.5%)	0.838	14 (12.5%)	9 (22.0%)	0.147
	Personality disorder	11 (9.0%)	4 (8.0%)	0.830	19 (8.0%)	6 (7.3%)	0.846	13 (11.6%)	6 (14.6%)	0.615
Manic/hypomanic polarity at onset		75 (61.5%)	31 (62.0%)	0.949	128 (53.8%)	43 (52.4%)	0.834	65 (58.0%)	17 (41.5%)	0.069
Predominant polarity	Manic	42 (34.4%)	23 (46.0%)	0.155	87 (36.6%)	38 (46.3%)	0.117	27 (24.1%)	7 (17.1%)	0.354
	Depressive	12 (9.8%)	5 (10.0%)	0.974	44 (18.5%)	12 (14.6%)	0.428	9 (8.0%)	18 (43.9%)	<0.001*
Recurrent episode (≥3)		83 (68.0%)	26 (52.0%)	0.048*	145 (60.9%)	55 (67.1%)	0.321	83 (74.1%)	38 (92.7%)	0.012*
Seasonal pattern		20 (16.4%)	6 (12.0%)	0.465	28 (11.8%)	16 (19.5%)	0.079	19 (17.0%)	4 (9.8%)	0.269
Rapid cycling		8 (6.6%)	1 (2.0%)	0.223	7 (2.9%)	1 (1.2%)	0.389	6 (5.4%)	1 (2.4%)	0.444
Psychotic symptom		45 (36.9%)	20 (40.0%)	0.702	81 (34.0%)	27 (32.9%)	0.855	35 (31.3%)	12 (29.3%)	0.814
Mixed episode		26 (21.3%)	3 (6.0%)	0.015*	44 (18.5%)	9 (11.0%)	0.115	20 (17.9%)	7 (17.1%)	0.910
Suicide attempt		16 (13.1%)	3 (6.0%)	0.176	21 (8.8%)	8 (9.8%)	0.800	17 (15.2%)	7 (17.1%)	0.775
Concomitant medication	antipsychotics	103 (84.4%)	41 (82.0%)	0.696	209 (87.8%)	69 (84.1%)	0.396	85 (75.9%)	31 (75.6%)	0.971
	antidepressants	29 (23.8%)	7 (14.0%)	0.153	43 (18.1%)	19 (23.2%)	0.313	50 (44.6%)	25 (61.0%)	0.073
Comorbid metabolic abnormalities	one or more abnormalities	32 (26.2%)	15 (30.0%)	0.614	54 (22.7%)	25 (30.5%)	0.158	34 (30.4%)	12 (29.3%)	0.896
	diabetes mellitus	16 (13.1%)	10 (20.0%)	0.252	30 (12.6%)	11 (13.4%)	0.850	14 (12.5%)	6 (14.6%)	0.729
	hypertension	19 (15.6%)	9 (18.0%)	0.696	25 (10.5%)	13 (15.9%)	0.197	19 (17.0%)	6 (14.6%)	0.730
	dyslipidemia	15 (12.3%)	7 (14.0%)	0.761	34 (14.3%)	15 (18.3%)	0.385	17 (15.2%)	8 (19.5%)	0.521

ISR: insufficient response, GR: good response

*p<0.05

### Predictors of response

A diagnosis of BD II (odds ratio [OR], 8.868; 95% confidence interval [CI], 1.123–70.046; p = 0.038), a comorbid alcohol/substance use disorder (OR, 4.238; 95% CI, 1.154–15.566; p = 0.030), and a history of mixed episodes (OR, 4.363; 95% CI, 1.191–15.985; p = 0.026) were significant predictors of LIT-ISR (Table 4). When diagnoses of BD II, comorbid alcohol/substance use disorder, predominant polarity, manic/hypomanic polarity at onset, or recurrent episodes were included as covariates in the analysis, a depressive predominant polarity significantly predicted LMT-GR (OR, 8.586; 95% CI, 2.767–26.644; p < 0.001). There were no significant predictors for the VAL treatment response when seasonal pattern was included as a covariate.

**Table 4:** Models of multivariate logistic regression.

Predictors for insufficient response to lithium treatment	Significance	OR	95% CI	
			Lower	Upper
Sex (male)	0.120	1.773	0.861	3.654
Age	0.262	1.016	0.989	1.043
Bipolar II disorder	0.038	8.868	1.123	70.046
Comorbid alcohol/substance use disorder	0.030	4.238	1.154	15.566
Recurrent episode (3 or more)	0.119	1.782	0.862	3.684
Mixed episode	0.026	4.363	1.191	15.985
Predictors for good response to valproate treatment				
Sex (male)	0.500	1.192	0.716	1.984
Age	0.578	1.005	0.988	1.022
Seasonal pattern	0.069	1.879	0.952	3.708
Predictors for good response to lamotrigine treatment				
Sex (male)	0.487	1.372	0.563	3.342
Age	0.412	1.012	0.983	1.042
Bipolar II disorder	0.959	1.027	0.372	2.837
Comorbid alcohol/substance use disorder	0.998	527953214.700	0.000	
Manic/Hypomanic polarity at onset	0.203	0.571	0.241	1.353
Depressive predominant polarity	<0.001	8.586	2.767	26.644
Recurrent episode (3 or more)	0.064	3.911	0.922	16.589
Concomitant use of antidepressant	0.657	0.817	0.335	1.993

OR; odds ratio, CI; confidence interval

## Discussion

The primary objective of the present study was to identify factors associated with long-term treatment responses in a large clinically representative sample of BD patients. Of the 625 BD patients who had been treated with mood stabilizers for at least 6 months, 172 were treated with LIT, 320 were treated with VAL, and 153 were treated with LMT. In contrast to previous studies [12–15, 27, 28], the present study included three agents commonly used for the treatment of BD (LIT, VAL, and LMT) and examined the associations between treatment response and the clinical characteristics of the subjects. These characteristics included factors that were recently found to be associated with the treatment response to BD, such as predominant polarity [29–31] and comorbid metabolic abnormalities [32, 33].

The present findings indicated that a comorbid alcohol/substance use disorder and a history of mixed episodes were associated with a poor response to LIT, as previously described [12, 15]. Additionally, the present study found that a diagnosis of BD II was associated with a poor response to LIT, which is consistent with other recent findings [15]. Recent guidelines for the management of patients with BD recommend LIT as a second-line agent for BD II depression based on studies showing that LIT is not superior to placebo for this disorder [8]. BD II patients experience more depressive symptoms than BD I patients [34] and present with significantly more depressive symptoms [35]; the relatively low effectiveness of LIT for BD depression may contribute to this result. However, controversy remains regarding the relationship between a diagnosis of BD II and the LIT response because some studies show that BD II patients have a better response to LIT [36, 37], whereas others show that diagnosis is unrelated to the LIT response [12]. It is also important to consider the serum levels of LIT when assessing the treatment response. Studies that reported a negative association between a diagnosis of BD II and a poor response to LIT did not evaluate the LIT concentrations [38] or recorded an extremely wide range (0.3–1.0 mmol/L) of serum LIT levels [39]. On the other hand, studies with appropriate LIT serum levels reported the relatively poor effectiveness of LIT for the long-term treatment of BD II [40–42].

Another important finding of the present study was that a depressive predominant polarity significantly predicted a good response to LMT. This result supports the recommendations of recent treatment guidelines that suggest LMT for the maintenance phase of patients with depressive predominant polarity [8, 43, 44]. Predominant polarity is of great importance for the formulation of management goals and confers a significant amount of information regarding treatment choices for patients, as previously suggested [45]. The usefulness of LMT for patients with depressive predominant polarity was demonstrated in a recent naturalistic study [46]. These authors also identified three prescription strategies for BD patients: 1) the "anti-manic stabilization package", which includes treatments with anti-manic mechanisms of action for predominantly manic/psychotic BD I patients; 2) the "anti-depressive stabilization package", which groups predominantly depressed patients; and 3) the "anti-bipolar II package", which groups BD II patients with a depressive predominant polarity, melancholic features, and higher rates of suicide behaviors. It is noteworthy that the “anti-depressive stabilization package” includes the prescriptions of LMT and other atypical antipsychotics, such as quetiapine, which has proven efficacy for the depressive phases of BD.

Previous studies have also investigated other possible predictors of the treatment response to mood stabilizers, including age at onset, number of previous episodes, rapid cycling, psychotic features, and family history of BD [12]. None of these characteristics had a significant association with treatment response in the present study and should be analyzed in future studies with larger sample sizes. Furthermore, recent evidence suggests that BD patients with comorbid metabolic dysregulation might exhibit greater probabilities of a chronic course of illness and treatment resistance compared to BD patients without metabolic issues [32, 47]. However, in the present study, comorbid metabolic abnormalities, such as dyslipidemia, diabetes mellitus, and hypertension, were not associated with the long-term treatment response to mood stabilizers. It is important to note that the diagnoses of metabolic abnormalities in the present study were extracted from available medical records, which might have underestimated the presence of metabolic conditions.

The present study has several possible limitations that should be considered when interpreting the findings. First, the retrospective assessment of the long-term response to treatment may have introduced a recall bias and/or reviewer bias. Additionally, diagnoses of BD I, BD II, and other psychiatric comorbidities were made clinically. Second, the associations observed in the present study might have been influenced by uncontrolled confounders, such as selection, dosage, and serum levels of index medications, because much of the clinical information was obtained during natural clinical practice. Moreover, the rate of patients receiving antipsychotics did not differ between insufficient response group and good response group. However, it is not still possible to rule out the possibility that atypical antipsychotics provided mood stabilizing effect. Third, the relatively small sample sizes of BD patients in each group resulting from low long-term treatment success or high retention rates could have caused type-II errors. A large proportion of BD patients failed to maintain index medication for more than 6 months, and thus, were ineligible for this study. Additionally, the prevalence of comorbid anxiety disorder in the LIT-ISR group (15.6%) was nearly double that of the LIT-GR group (8.0%); this difference did not reach statistical significance. Finally, the present study included Korean patients only. Moreover, demographic and clinical characteristics of patients who were excluded from the study were not investigated, which could have caused potential bias. Thus, the findings should be interpreted cautiously.

## Conclusion

Despite these limitations, the present study identified several associations between clinical factors and the long-term responses to mood stabilizers commonly used for the treatment of BD. Although previous studies already investigated factors predicting good treatment response or mood stabilizing effect of mood stabilizers, these studies investigated predictors of individual mood stabilizers only. In contrast, our results provided additional information by including three mood stabilizers (LMT, LIT, and VAL) and investigating their long-term mood stabilizing effects. The results from this naturalistic study has enable us to directly compare characteristics of the three mood stabilizers. By doing so, the study improved the current understanding of how clinical characteristics of BD disorder patients might predict treatment response to a specific drug, and it may provide a more valid response phenotype for future genetic and other studies.

## Supporting information

S1 Dataset (XLSX)
